# Phenotypic Variants of Bacterial Colonies in Microbiological Diagnostics: How Often Are They Indicative of Differing Antimicrobial Susceptibility Patterns?

**DOI:** 10.1128/Spectrum.00555-21

**Published:** 2021-09-22

**Authors:** Neele J. Froböse, Franziska Schuler, Alexander Mellmann, Marc T. Hennies, Evgeny A. Idelevich, Frieder Schaumburg

**Affiliations:** a Institute of Medical Microbiology, University Hospital Münster, Münster, Germany; b Institute for Hygiene, University Hospital Münster, Münster, Germany; c Friedrich Loeffler-Institute of Medical Microbiology, University Medicine Greifswald, Greifswald, Germany; University of West London

**Keywords:** antimicrobial susceptibility testing, polyclonal infection, antimicrobial stewardship, phenotypic variant, microbiological diagnostics

## Abstract

Phenotypic variants (PV) are colonies of the same species in the same specimen with different morphological features. It is controversial whether antimicrobial susceptibility testing (AST) should be done for all PV. The objectives of this study were to quantify the proportion of differing antimicrobial susceptibility patterns (dASP) among PV and to identify species and antimicrobial compounds that are mostly affected. All PV from routine diagnostics (University Hospital Münster, Germany; 1 September 2019 to 31 August 2020) were subjected to species identification (matrix-assisted laser desorption ionization–time of flight mass spectrometry [MALDI-TOF MS]) and AST (Vitek 2). To assess the dASP, only antimicrobial agents were considered for which Vitek-derived MIC were available (interpreted according to the EUCAST clinical breakpoints). The categorical agreement (CA; agreement with the AST categories S [susceptible, standard dosing regimen], I [susceptible, increased exposure], R [resistant]) of the PV was calculated. The PV of Escherichia coli (*n* = 260), Pseudomonas aeruginosa (*n* = 86), Klebsiella pneumoniae (*n* = 47), Enterobacter cloacae complex (*n* = 45), and Staphylococcus aureus (*n* = 38) were included. The median CA was 95% (range, 80 to 100%, depending on the species). The colony characteristics (e.g., form/size, color, margin, hemolysis) were not indicative for dASP. PV showed a high categorical agreement in the AST categories. This observation supports a test strategy to perform AST for only one colony of PV.

**IMPORTANCE** Phenotypic variants of bacteria are frequent in routine diagnostics and can display differing antimicrobial susceptibility patterns. We found that the likelihood of different antimicrobial susceptibility is low among PV. To save laboratory resources, only one isolate per PV could be tested to guide the antimicrobial treatment of patients.

## INTRODUCTION

In routine diagnostics, phenotypic variants (PV) of the same bacterial species in the same specimen are considered colonies with different morphological features. In addition to size, shape, color, margin, elevation, and texture (1), colonies of PV can be differentiated based on hemolysis or the ability to ferment lactose as generic distinctive features in microbiological diagnostics. PV are attributed to genetic differences (e.g., enzyme/toxin expression, pigment production) (2) and/or different properties, such as motility or virulence (e.g., hypervirulent hypermucoviscous Klebsiella pneumoniae) (3). In addition, PV can be linked with antimicrobial resistance: rough small-colony variants of Pseudomonas aeruginosa in cystic fibrosis (CF) patients are often aminoglycoside resistant (4), small-colony variants of Staphylococcus aureus may be more resistant than the normal phenotype (5), and most of hypermucoviscous K. pneumoniae from Asia are susceptible toward the majority of antimicrobials (3). There are sporadic case reports on polyclonal infections (i.e., endocarditis caused by coagulase-negative staphylococci) with differing antimicrobial susceptibility patterns (dASP) of the PV (6, 7). However, it is unclear to what extent this applies to routine diagnostics and whether we have to test all the PV of one species in one specimen in order not to miss therapeutically relevant antimicrobial resistance. Therefore, the objectives of this study were to quantify the proportion of dASP among PV and to identify the species/antimicrobial agents that are mostly affected.

## RESULTS

In total, 1,087 isolates covering 22 species from 537 individual patients were enrolled. In the majority of cases, we observed pairs of PV (*n* = 525), followed by triplets (*n* = 11) and one quartet. Of these 537 PV, 61 (including 17 species) were removed due to small sample sizes (case count, *n* < 30 per species; Fig. S1 in the supplemental material).

The final data set included 476 PV; 49.8% (*n* = 237) of the patients from which the PV originated were female, and the median age was 62.3 years (range, 0 to 95.1). The specimens were from infection (*n* = 409 [212 urine samples, 84 swabs, 50 respiratory secretions, 27 puncture fluids, 36 others]) or colonization (*n* = 67). PV were detected among pairs (*n* = 467), triplets (*n* = 8), and quartets (*n* = 1) of morphologically distinct colonies. The included PV comprised the following species: Escherichia coli (*n* = 260), P. aeruginosa (*n* = 86), K. pneumoniae (*n* = 47), Enterobacter cloacae complex (*n* = 45), and S. aureus (*n* = 38). Taken together, the median categorical agreement (CA) for all species was 95% (range, 80 to 100%, depending on the species; Table 1).

**TABLE 1:** Categorical agreement of antimicrobial susceptibility testing for phenotypic variants^*a*^

Bacterial species of PV	Antimicrobial agent (no. of tested isolates)	Antimicrobial susceptibility of PV (no. of isolates [%]):			Categorical agreement of PV (no. of isolates/total no. of isolates [%])	Differing antimicrobial susceptibility test results within the ATU among PV (no. of ATU/no. of categorical disagreements)
		S	I	R		
Escherichia coli	Ampicillin (524)	210 (40.1)		314 (59.9)	220/260^*b*^ (84.6)	NA
	Ampicillin-sulbactam (212)^*c*^	96 (45.3)		116 (54.7)	88/104^*b*^ (84.6)^*c*^	NA
	Amoxicillin-clavulanic acid (312)^*d*^	155 (49.7)		157 (50.3)	133/156^*b*^ (85.3)^*d*^	NA
	Piperacillin-tazobactam (524)	444 (84.7)	2 (0.4)	78 (14.9)	243/260^*b*^ (93.5)	3/17
	Cefuroxime (524)	150 (28.6)	234 (44.7)	140 (26.7)	220/260^*b*^ (84.6)	NA
	Cefuroxime-axetil (524)	383 (73.1)		141 (26.9)	223/260^*b*^ (85.8)	NA
	Cefotaxime (524)	429 (81.9)	1 (0.2)	94 (17.9)	242/260^*b*^ (93.1)	NA
	Cefpodoxime (524)	396 (75.6)		128 (24.4)	230/260^*b*^ (88.5)	NA
	Ceftazidime (524)	427 (81.5)	6 (1.2)	91 (17.4)	241/260^*b*^ (92.7)	NA
	Cefepime				143/260^*b*^ (91.7)^*d*^	NA
	Ertapenem (524)	523 (99.8)		1 (0.2)	259/260^*b*^ (99.6)	NA
	Imipenem (212)^*c*^	212 (100)			104/104^*b*^ (100)^*c*^	NA
	Meropenem (524)	524 (100)			260/260^*b*^ (100)	NA
	Gentamicin (212)^*c*^	195 (92.0)		17 (8.0)	99/104^*b*^ (95.2)^*c*^	NA
	Ciprofloxacin (524)	370 (70.6)	19 (3.6)	135 (25.8)	225/260^*b*^ (86.5)	12/35
	Levofloxacin (312)^*d*^	201 (64.4)	37 (11.9)	74 (23.7)	136/156^*b*^ (87.2)^*d*^	NA
	Moxifloxacin (212)^*c*^	133 (62.7)		79 (37.3)	91/104^*b*^ (87.5)^*c*^	NA
	Trimethoprim (312)^*d*^	189 (60.6)		123 (39.4)	140/156^*b*^ (89.7)^*d*^	NA
	Trimethoprim-sulfamethoxazole (524)	348 (66.4)		176 (33.6)	229/260^*b*^ (88.1)	NA
	Tigecycline (212)^*c*^	201 (94.8)		11 (5.2)	98/104^*b*^ (94.2)^*c*^	NA
	Nitrofurantoin (312)^*d*^	306 (98.1)		6 (1.9)	154/156^*b*^ (98.7)^*d*^	NA
	Mecillinam (312)^*d*^	282 (90.4)		30 (9.6)	142/156^*b*^ (91.0)^*d*^	NA
	Fosfomycin (312)	308 (98.7)		4 (1.3)	156/156^*b*^ (100)^*d*^	NA
Pseudomonas aeruginosa	Piperacillin (176)	0 (0)	129 (73.3)	47 (26.7)	83/86^*e*^ (97)	NA
	Piperacillin-tazobactam (176)	0 (0)	130 (73.9)	46 (26.1)	82/86^*e*^ (95)	NA
	Ceftazidime (176)	0 (0)	137 (77.8)	39 (22.2)	79/86^*e*^ (92)	NA
	Cefepime (176)	0 (0)	140 (79.6)	30 (17.0)	80/86^*e*^ (93)	NA
	Aztreonam (176)	0 (0)	138 (78.4)	38 (21.6)	82/86^*e*^ (95)	NA
	Imipenem (176)	0 (0)	140 (79.6)	30 (17.0)	80/86^*e*^ (93)	NA
	Meropenem (176)	138 (78.4)	17 (9.7)	21 (11.9)	79/86^*e*^ (92)	NA
	Amikacin (176)	141 (80.1)	32 (18.2)	3 (1.7)	82/86^*e*^ (95)	NA
	Tobramycin (176)	141 (80.1)	31 (17.6)	4 (2.3)	84/86^*e*^ (98)	NA
	Ciprofloxacin (176)	0 (0)	157 (89.2)	19 (10.8)	81/86^*e*^ (94)	NA
	Colistin (176)	175 (99.4)	0 (0)	1 (0.6)	85/86^*e*^ (99)	1/1
Klebsiella pneumoniae	Ampicillin (94)			94 (100)	47/47^*f*^ (100)	NA
	Ampicillin-sulbactam (44)^*g*^	27 (61)		17 (39)	21/22^*f*^ (95)^*g*^	NA
	Amoxicillin-clavulanic acid (50)^*h*^	27 (54)		23 (46)	20/25^*f*^ (80)^*h*^	NA
	Piperacillin-tazobactam (94)	59 (63)	13 (14)	22 (23)	42/47^*f*^ (89)	0/5
	Cefuroxime (94)	18 (19)	45 (48)	31 (33)	44/47^*f*^ (94)	NA
	Cefuroxime-axetil (94)	59 (63)	4 (4)	31 (33)	44/47^*f*^ (94)	NA
	Cefotaxime (94)	88 (94)		6 (6)	47/47^*f*^ (100)	NA
	Cefpodoxime (94)	82 (87)		12 (13)	43/47^*f*^ (91)	NA
	Ceftazidime (94)	78 (83)	7 (8)	9 (10)	42/47^*f*^ (89)	NA
	Cefepime (50)^*h*^	46 (92)	4 (8)		23/25^*f*^ (92)^*h*^	NA
	Ertapenem (94)	89 (95)		5 (5)	46/47^*f*^ (98)	NA
	Imipenem (44)^*g*^	42 (95)	2 (5)		22/22^*f*^ (100)^*c*^	NA
	Meropenem (94)	93 (99)		1 (1)	46/47^*f*^ (98)	NA
	Gentamicin (44)^*g*^	44 (100)			22/22^*f*^ (100)^*c*^	NA
	Ciprofloxacin (94)	82 (87)		12 (13)	47/47^*f*^ (100)	0/0
	Levofloxacin (50)^*h*^	40 (80)	6 (12)	4 (8)	25/25^*f*^ (100)^*h*^	NA
	Moxifloxacin (44)^*g*^	34 (77)		10 (23)	22/22^*f*^ (100)^*c*^	NA
	Trimethoprim (50)^*h*^	41 (82)	2 (4)	7 (14)	24/25^*f*^ (96)^*h*^	NA
	Trimethoprim-sulfamethoxazole (94)	79 (84)		15 (16)	46/47^*f*^ (98)	NA
	Fosfomycin (50)^*h*^	35 (70)		15 (30)	24/25^*f*^ (96)^*h*^	NA
Enterobacter cloacae complex	Piperacillin-tazobactam (91)	4 (4)	4 (4)	29 (32)	41/45^*i*^ (91)	2/4
	Cefotaxime (91)	63 (69)		28 (31)	42/45^*i*^ (93)	NA
	Cefpodoxime (91)	7 (8)		84 (92)	40/45^*i*^ (89)	NA
	Ceftazidime (91)	60 (66)	3 (3)	28 (31)	41/45^*i*^ (91)	NA
	Cefepime (26)^*j*^	18 (69)		8 (30)	11/13^*i*^ (84.6)^*j*^	NA
	Ertapenem (91)	80 (88)		11 (12)	42/45^*i*^ (93)	NA
	Imipenem (65)^*k*^	65 (100)			32/32^*i*^ (100)^*k*^	NA
	Meropenem (91)	91 (100)			45/45^*i*^ (100)	NA
	Gentamicin (65)^*d*^	64 (98)		1 (2)	31/32^*i*^ (97)^*k*^	NA
	Ciprofloxacin (91)	83 (91)	1 (1)	7 (8)	43/45^*i*^ (96)	1/2
	Levofloxacin (26)^*j*^	19 (73)	5 (19)	2 (8)	12/13^*i*^ (92)^*j*^	NA
	Moxifloxacin (65)^*k*^	62 (95)		3 (5)	31/32^*i*^ (97)^*k*^	NA
	Trimethoprim (26)	15 (58)		11 (42)	12/13^*i*^ (92)^*j*^	NA
	Trimethoprim-sulfamethoxazole (91)	75 (82)		16 (18)	45/45^*i*^ (100)	NA
	Fosfomycin (26)	17 (65)		9 (35)	12/13^*i*^ (92)^*j*^	NA
Staphylococcus aureus	Benzylpenicillin (77)	27 (35)	NA	50 (65)	35/38^*l*^ (92)	NA
	Oxacillin (77)	70 (91)	NA	7 (9)	38/38^*l*^ (100)	NA
	Erythromycin (77)	58 (75)	0 (0)	19 (25)	34/38^*l*^ (90)	NA
	Gentamicin (77)	76 (99)	0 (0)	1 (1)	37/38^*l*^ (97)	NA
	Levofloxacin (77)	37 (48)	32 (42)	8 (10)	37/38^*l*^ (97)	NA
	Clindamycin (77)	64 (83)	0 (0)	13 (17)	36/38^*l*^ (95)	NA
	Vancomycin (77)	77 (100)	NA	0 (0)	38/38^*l*^ (100)	NA
	Teicoplanin (77)	77 (100)	NA	0 (0)	38/38^*l*^ (100)	NA
	Linezolid (77)	77 (100)	NA	0 (0)	38/38^*l*^ (100)	NA
	Daptomycin (77)	77 (100)	NA	0 (0)	38/38^*l*^ (100)	NA
	Trimethoprim-sulfamethoxazole (77)	77 (100)	0 (0)	0 (0)	38/38^*l*^ (100)	NA
	Tetracycline (77)	72 (94)	0 (0	5 (6)	36/38^*l*^ (95)	NA
	Tigecycline (77)	0 (0)	NA	77 (100)	38/38^*l*^ (100)	NA
	Rifampin (77)	77 (100)	0 (0	0 (0)	38/38^*l*^ (100)	NA
	Fusidic acid (77)	74 (96)	NA	3 (4)	37/38^*l*^ (97)	NA
	Fosfomycin (77)	75 (97)	NA	2 (3)	36/38^*l*^ (95)	NA

a PV, phenotypic variant; ATU, area of technical uncertainty (according to EUCAST); NA, not applicable.

b 256 pairs, 4 triplets.

c Applies to 104 patients with non-urine samples (a differing set of antimicrobial agents was used for antimicrobial susceptibility testing of E. coli from urine samples; see Materials and Methods for details).

d Applies to 156 patients with urine samples (a differing set of antimicrobial agents was used for antimicrobial susceptibility testing of E. coli from urine samples; see Materials and Methods for details).

e 83 pairs, 2 triplets, 1 quartet.

f 47 pairs.

g Applies to 22 patients with non-urine samples (a differing set of antimicrobial agents was used for antimicrobial susceptibility testing of K. pneumoniae from urine samples; see Materials and Methods for details).

h Applies to 25 patients with urine samples (a differing set of antimicrobial agents was used for antimicrobial susceptibility testing of K. pneumoniae from urine samples; see Materials and Methods for details).

i 44 pairs, 1 triplet.

j Applies to 13 patients with urine samples (a differing set of antimicrobial agents was used for antimicrobial susceptibility testing of E. cloacae complex from urine samples; see Materials and Methods for details).

k Applies to 32 patients with non-urine samples (a differing set of antimicrobial agents was used for antimicrobial susceptibility testing of E. cloacae complex from urine samples; see Materials and Methods for details).

l 37 pairs, 1 triplet.

The PV of E. coli (*n* = 260) were differentiated based on the margin (*n* = 101; 38.9%), fermentation of lactose (*n* = 70; 26.9%), color (*n* = 42; 16.2%), size/shape (*n* = 31; 11.9%), and hemolysis (*n* = 16; 6.2%).

A dASP was detected in 116 (44.6%) PV of E. coli. The median number of antimicrobial agents affected by dASP was 3 (range, 1 to 13). The CA was lowest for penicillins with/without beta-lactamase inhibitors (84.6 to 85.3%) and quinolones (86.5 to 87.5%; Table 1). The highest likelihood for dASP was found in PV with variance in lactose fermentation (e.g., lactose positive versus lactose negative; odds ratio [OR] = 1.8; 95% confidence interval [CI], 1.0 to 3.2; *P* = 0.07). However, none of the categories to describe PV were significantly associated with and therefore predictive of a dASP.

The PV in P. aeruginosa (*n* = 86) were based on margin (*n* = 46; 54%), color (*n* = 27; 31%), or colony size/shape (*n* = 13; 15%). Twenty-one P. aeruginosa PV (24%) showed a dASP. The median number of dASP among the PV was 0 (range, 0 to 6). The CA for the tested antimicrobial agents was 92 to 97% (Table 1). None of the categories that we used to describe the PV of P. aeruginosa were associated with dASP (*P* > 0.5).

The PV of K. pneumoniae (*n* = 47) were distinguished according to the fermentation of lactose (*n* = 16; 34%), margin (*n* = 12; 26%), color (*n* = 13; 28%), and shape/size (*n* = 6; 13%). Of the 47 PV, 16 (34%) had a dASP. Of these, the median number of antimicrobial agents that caused dASP was 2 (range, 1 to 5). Apart from amoxicillin-clavulanic acid, piperacillin-tazobactam, and cefpodoxime, the CA was ≥90% for the tested antimicrobial agents (Table 1). None of the categories that defined PV were indicative for dASP (*P* > 0.4).

The PV of the E. cloacae complex (*n* = 45) were differentiated based on the fermentation of lactose (*n* = 19; 42%), margin (*n* = 16; 37%), color (*n* = 6; 13%), and colony size/shape (*n* = 4; 9%). Of these, 33% (*n* = 15) had a dASP. The median number of antimicrobial agents that caused dASP was 1 (range, 1 to 7). The lowest proportions of CA had cefepime (84%) and cefpodoxime (89%; Table 1). None of the morphological characteristics of the E. cloacae complex PV (i.e., fermentation of lactose, margin, color, size/shape) were associated with the presence of dASP (*P* > 0.3).

The PV of S. aureus (*n* = 38) were related to hemolysis (*n* = 25; 66%), color (*n* = 9; 24%), or colony size/shape (*n* = 4; 11%). Eight PV of S. aureus (21%) had dASP. The median number of dASP among PV was 0 (range, 0 to 6). The CA for individual agents ranged between 90 and 100% (Table 1). None of the categories that we applied to distinguish PV (e.g., hemolysis) was significantly associated with the presence of dASP (*P* = 0.06).

The majority of dASP among PV could not be explained by areas of technical uncertainty (ATUs). Only a notable proportion of differing test results for ciprofloxacin susceptibility testing in E. coli could be traced back to ATUs (34%; 12/35; Table 1). The number of antimicrobial agents with differing test results was comparable for PV from almost all species. Only the median number (range) of antimicrobial agents with differing test results were higher in E. coli compared to P. aeruginosa (3 [1 to 13] versus 0 [0 to 6]; *P* = 0.006). The number of differing test results was comparable between PV from infection and colonization (*P* = 0.2).

## DISCUSSION

We assessed the proportion of dASP in the PV of major species (i.e., E. coli, E. cloacae complex, K. pneumoniae, P. aeruginosa, S. aureus) from routine diagnostics and found that the CA of AST results between PV was high (median, 95%; range, 80 to 100%, depending on the species).

In the majority of cases, we identified PV in pairs (467 out of 476 in total) of one species in one specimen. However, particularly in chronic infections or low-grade infections, up to five PV of the same species were found in one patient (8).

Looking only at the proportion of dASP is short-sighted and might be misleading, as the proportion of dASP can be up to 44.6% (E. coli); however, this does not reflect the high CA of AST results in PV when looking at individual antimicrobial agents (Table 1). Although PV can be associated with differing antimicrobial susceptibility patterns (3–7, 9), this does not apply for the vast majority of PV in routine diagnostics in our setting (Table 1). The most likely reason is that PV do not represent different genotypes but different expression profiles of the same clone harboring the same set of resistance genes (8). The high proportion of CA among PV, particularly for therapeutically relevant agents such as oxacillin in S. aureus (100%; Table 1) or carbapenemes for the E. cloacae complex (100%; Table 1), can justify testing only one isolate if several PV are detected. However, this decision should take into account the local resistance data and prescribing policies, as some relevant antimicrobial agents can show low proportions of CA (e.g., 80% for amoxicillin-clavulanic acid in K. pneumoniae).

The limitations of our study are as follows. (i) We did not perform genotyping (e.g., whole-genome sequencing) and are therefore unable to state whether PV were clonally related and to what extent clonality had an impact on the amount of CA. (ii) Serial passages were not performed to assess the stability of PV and whether the AST pattern can revert. (iii) The study population represents patients from a tertiary care hospital with higher exposure to antimicrobial agents. Our results might therefore not be applicable to settings of primary or secondary care levels. (iv) The influence of chronic infections on the detection of PV could not be excluded, as we did not verify underlying chronic infections. As most PV occurred in pairs, it can be assumed that chronic infections were rare among our samples, because chronic infections are usually associated with more PV (e.g., 5 variants) (8).

## CONCLUSION

The PV of one species from one specimen showed a high categorical agreement in the AST categories S (susceptible, standard dosing regimen), I (susceptible, increased exposure), and R (resistant). Thus, it appears sufficient to select only one colony of PV for antimicrobial susceptibility testing.

## MATERIALS AND METHODS

### Ethics.

Ethical approval was obtained from the institutional review board (IRB, Ethikkommission der Westfälischen Wilhelmsuniversität Münster, 2020-353-f-S). The IRB granted a waiver for obtaining signed written informed consent from patients.

### Study design.

This cross-sectional study was performed in a routine laboratory (Institute of Medical Microbiology, University Hospital Münster, Germany) between 1 September 2019 and 31 August 2020. The inclusion criteria were PV from colonization and infection. The exclusion criteria were as follows: (i) follow-up PV of the same species from the same patient; (ii) CF patients, due to a *per se* high prevalence of PV in isolates from respiratory specimens; and (iii) species for which EUCAST clinical breakpoints are not yet available.

The decision to send a specimen for microbiological analysis was solely based on the physicians’ judgment. For patients, we recorded the age, sex, and specimen sent for analysis. The specimens were stratified into “colonization” (e.g., screening swabs to detect colonization with methicillin-resistant Staphylococcus aureus in nasal swabs or colonization with multidrug-resistant Gram-negative bacteria in anal swabs) or “infection” (e.g., blood culture, wound swabs, cerebrospinal fluid [CSF]).

### Microbiology.

**FIG 1:**
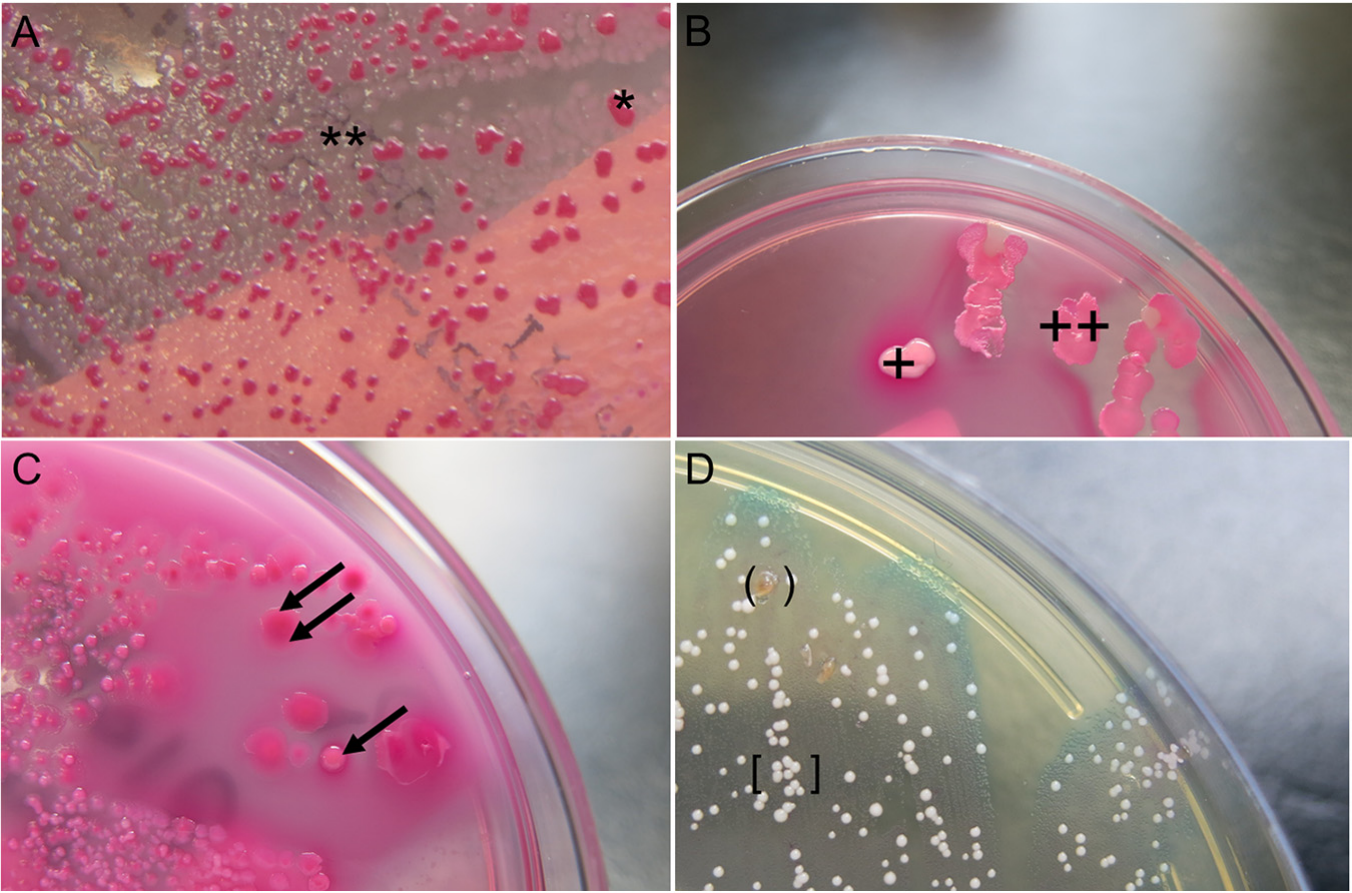
Categories of PV differentiation: (A) Escherichia coli differentiated by the ability to ferment lactose (lactose positive [*] versus lactose negative [**]) on MacConkey agar; (B) E. coli differentiated by form (round [+] versus flat [++]) on MacConkey agar; (C) E. coli differentiated by margin (smooth [two arrows] versus fringed [one arrow]) on MacConkey agar; (D) Citrobacter freundii differentiated by color (brown in parentheses versus white in square brackets) on extended spectrum beta-lactamase (ESBL) agar.

The specimens were cultured on standard or selective agar plates (see Table S1 in the supplemental material for the included media) and incubated at 35 ± 2°C under aerobic, anaerobic, or 5% CO_2_ conditions according to the category of the sample (Table S1 in the supplemental material). We defined PV as two (i.e., pairs) or more (i.e., triplets, quartets) colonies of the same species in the same specimen that are morphologically distinguishable on one agar plate. One clinical microbiologist or laboratory technician screened bacterial colonies during routine diagnostics for PV based on the categories form, color (e.g., on selective chromogenic agar), margin, hemolysis, or the ability to ferment lactose (Fig. 1) (1).

The species were identified by matrix-assisted laser desorption ionization–time of flight (MALDI-TOF) mass spectrometry (Microflex; Bruker, Bremen, Germany). Antimicrobial susceptibility testing (AST) was done using the Vitek 2 automated system (bioMérieux, Marcy l’Étoile, France) and interpreted using the EUCAST clinical breakpoints version 9.0 (2019) or 10.0 (2020) (10, 11). A standard set of AST Vitek 2 cards was used for AST of *Enterobacterales* from urine (AST-N387), *Enterobacterales* from other specimens (AST-N214), P. aeruginosa (AST-N248), Staphylococcus spp. (AST-P654), and *Enterococcus/*Streptococcus spp. (AST-P655, AST-ST03). Only colonies from primary cultures were used for AST. The laboratory successfully participates quarterly in external quality assessments and performs weekly quality controls for species identification and AST.

### Statistical analysis.

Species were considered in the final analysis for which case counts of PV reached ≥30. We attributed each PV to the category in which the respective isolates differed (form, color, margin, hemolysis, or the ability to ferment lactose). We compared the AST results of PV to identify differing antimicrobial susceptibility patterns (i.e., when the AST results of the PV variants differed in the interpretative category [S, I, R] for the tested antimicrobial agents). Antimicrobial agents were only considered if the Vitek 2-derived MICs were available. Intrinsic resistances were not considered in the assessment of dASP (12).

The CA (agreement in the AST categories “S,” “I,” “R”) among the two or more bacterial colonies defining one PV was calculated for each antimicrobial agent (Table 1). In case of dASP, we recorded whether the MIC of the affected antibiotic was in an area of technical uncertainty (ATU) as defined by EUCAST (11).

An association between the category of PV and the presence of dASP was assessed using a logistic regression model. The numbers of differing AST results was compared between the PV of different species using pairwise Wilcoxon rank sum tests and the Holm adjustment for multiple testing. Statistical analyses were conducted using R version 3.6.1 (significance level, 0.05; epiDisplay package, https://cran.r-project.org).
