# Supplementary material for: Phenotypic Variants of Bacterial Colonies in Microbiological Diagnostics: How Often Are They Indicative of Differing Antimicrobial Susceptibility Patterns?
Source: Microbiol Spectr. 2021 Sep 22;9(2):e00555-21. doi: 10.1128/Spectrum.00555-21 (PMC8557900; doi:10.1128/Spectrum.00555-21)
Supplement: SUPPLEMENTAL FILE 1 — Supplemental material. Download SPECTRUM00555-21_Supp_1_seq4.pdf, PDF file, 0.3 MB [file spectrum00555-21_supp_1_seq4.pdf]

Supplement

Table S1: Standard set of culture media for the bacterial analysis of clinical material

	Tissue	CSF[a]	Superficial swab	Deep-tissue swab	Blood culture	Puncture specimens	Respiratory specimens	Urine	Catheter
Columbia blood agar[c]	+	+	+	+		+	+	+	+
Chocolate agar[d]	+	+	+	+	+	+	+		
MacConkey agar[d]			+				+	+	
Schaedler agar[d]	+	+		+	+[b]	+			
Thioglyocolate broth[c]	+	+	+	+		+	+		
BHI broth[e]									+
Overall incubation time [days]	3	2	2	2	7 (14[f])	2	2	2	2
Aerobic incubation (35±2°C)	+		+	+		+	+	+	+
Aerobic incubation with 5% CO_2 (35±2°C)	+	+	+	+	+	+	+		
Anaerobic incubation (35±2°C)	+	+		+	+[b]	+			

[a]cerebrospinal fluid

[b]only in cases of positive anaerobic blood cultures

[c]BD, Heidelberg, Germany

[d]Thermofisher, Oxoid, Hennigsdorf, Germany

[e]bioMérieux, Marcy l’Étoile, France

[f]if endocarditis is suspected

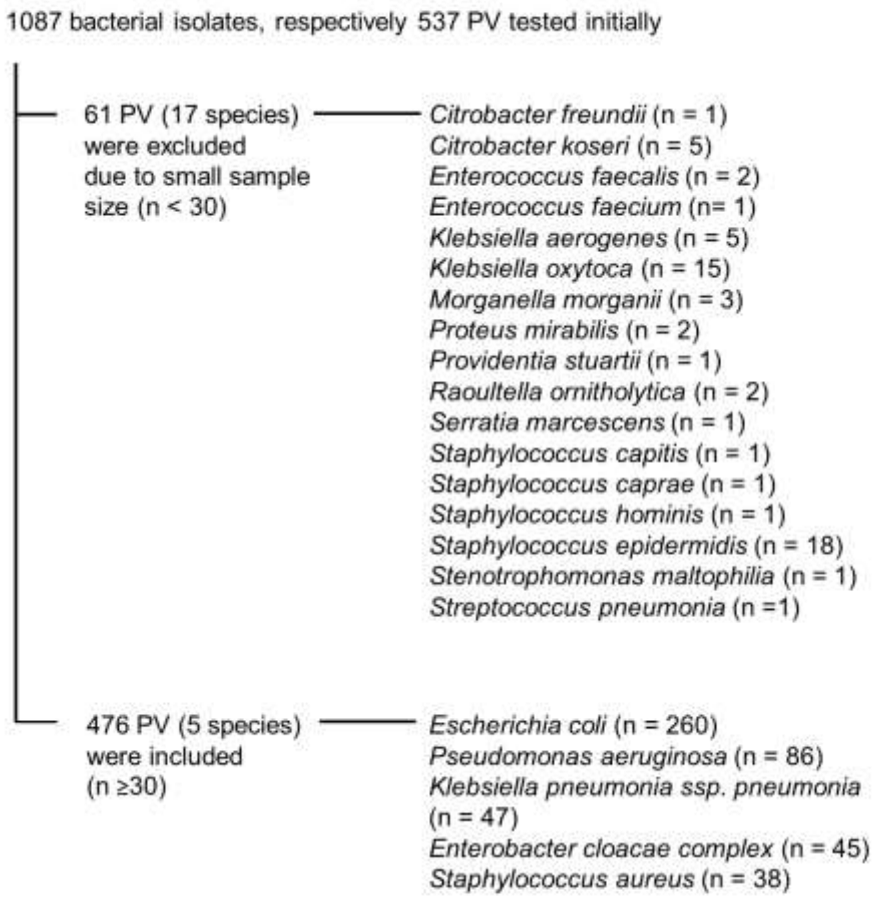

Figure S1: Outline of examined isolates: Species included and excluded of final analysis
